# Comparison of Pain Catastrophic Scale and Anxiety in Patients With Boxer’s Fracture and Other Types of Hand Fractures

**DOI:** 10.7759/cureus.35019

**Published:** 2023-02-15

**Authors:** Ömer Bozduman, Metin Yadigaroğlu, Ahmet E Okutan, Mustafa Süren, Berk Güçlü

**Affiliations:** 1 Orthopaedic Surgery, Samsun University, Samsun, TUR; 2 Emergency Medicine, Samsun University, Samsun, TUR; 3 Orthopaedics and Traumatology, Samsun University, Samsun, TUR; 4 Anesthesiology and Reanimation, Samsun University, Samsun, TUR; 5 Orthopaedics and Traumatology, Ufuk University, Ankara, TUR

**Keywords:** trauma, orthopedics, emergency medicine, boxer’s fracture, visual analogue scale, pain catastrophic scale

## Abstract

Objective:* *The Pain Catastrophic Scale (PCS) is generally associated with high and low post-recovery satisfaction and measures the pain perception of patients in the literature. This study aims to evaluate the association of deliberate (as in a fight or anger causing punching a wall) boxer’s fractures with catastrophic pain compared to accidental (as in a fall, accidental knocking it against a wall, etc.) fractures and evaluate the effect of anxiety about fracture union and functional recovery on clinical outcomes.

Materials and methods: A total of 62 male patients with metacarpal fractures, 31 as a result of deliberate punching (1st group) and 31 with metacarpal fractures as a result of an accident (2nd group), who applied to the emergency department or orthopedic clinic with the diagnosis of metacarpal fracture between January 2021 and October 2022, were included in the study. All patients were selected from patients who were followed up with conservative plaster/splint. The PCS scores of the patients were evaluated comparatively with the clinical results measured after at least six weeks.

Results:* *The mean age of the patients was 30.8 (18-50) in the 1st group and 34.8 (18-64) in the 2nd Group, and no statistically significant difference was found (p=0.274). While the median PCS score was 10.5 (interquartile range {IQR}: 12.3) for the 1st group, the median PCS score was 17.5 (IQR: 14.5) for the 2nd group, and the PCS score was statistically significantly lower in group 1 (p=0.009). While the median Visual Analogue Scale (VAS) value was 0 (IQR: 0.3) for the 1st group, the median VAS value was 1 (IQR: 2.0) for the 2nd group, and the VAS score was statistically significantly lower in the 1st group (p<0.001). While the median ‘quick disabilities of the arm, shoulder, and hand’ (Q-DASH) value was 0 (IQR:2.3) for the 1st group, the median Q-DASH value was 3.4 (IQR:6.3) for the 2nd group, and the Q-DASH score was statistically significantly lower in the 1st group (p=0.001). No significant difference was observed between the 1st and 2nd groups in terms of grip strength values (p=0.815).

Conclusion:The etiology of patients presenting with a boxer’s fracture should be well understood, and if necessary, these patients should be treated multidisciplinary, with psychiatric help. Better satisfaction can be achieved with lower results in patients whose PCS scoring system has lost its eigenvalue.

## Introduction

Metacarpal bone fractures account for approximately one-third of all hand fractures. Fifth metacarpal bone fractures account for up to 18.4% of these and it has been reported that more than 50% of metacarpal fractures are subcapital fractures [1-5]. These fifth metacarpal fractures are also referred to as “boxer’s fractures” [6,7], as they are often seen in males aged 15 to 55 years who exhibit aggressive behavior, involving punching a hard object [8,9].

Destructive thoughts about pain and pain anxiety are common protective responses, but if they last for a prolonged time, they can negatively affect recovery. The evaluation of destructive thoughts is an evaluation of coping with cognitive thoughts, such as magnifying the experience of pain, thinking about pain, being hopeless when experiencing pain, or feeling helpless [10].

Pain anxiety comprises several dimensions. Some subscales assess cognitive anxiety, pain-related anxiety, such as fear, escape, and avoidance, and physiological anxiety [11]. It has been shown that psychological factors are decisive in the clinical evaluation of surgical results and have an important role in the recovery of the patient [12].

This study aimed to evaluate the association of deliberate boxer’s fractures with catastrophic pain, compared to accidental fractures, and to evaluate the effect of anxiety concerning fracture union and functional recovery on clinical outcome.

## Materials and methods

The study was conducted by the Declaration of Helsinki and approved by the ethics committee of Samsun University Samsun Education and Research Hospital (No. 2022/7/10). The informed consent was waived due to the retrospective nature of the study and the fact that the assessment utilized anonymous research findings.

**Figure 1:**
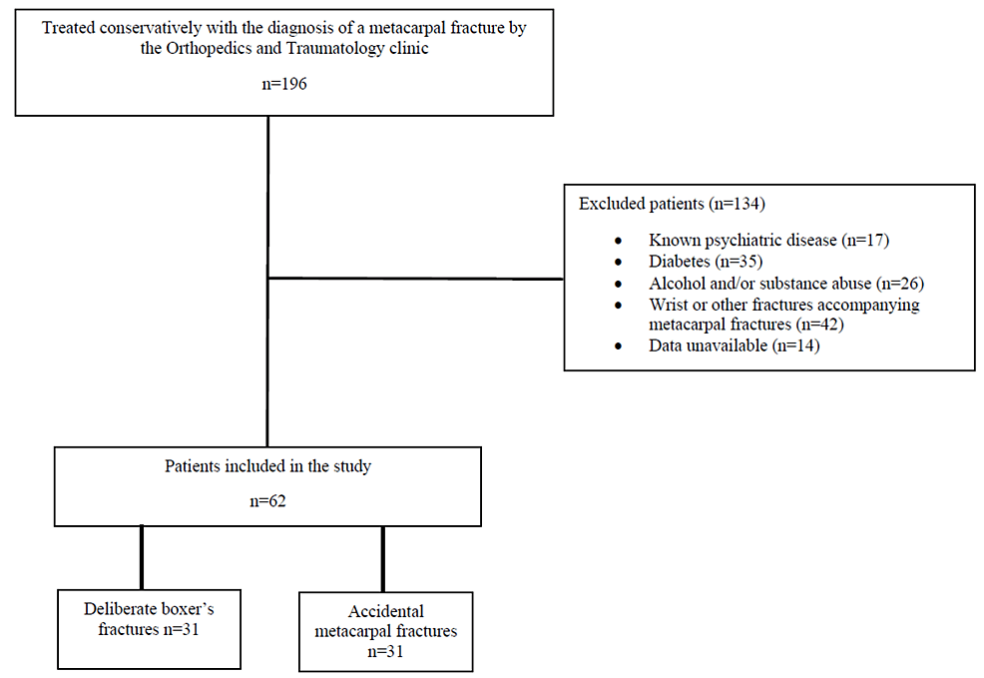
Study Flow Chart

In this study, the files of 196 patients who were treated conservatively with the diagnosis of a metacarpal fracture by the Orthopedics and Traumatology clinic, between January 2021 and October 2022, were retrospectively reviewed. Patients with known psychiatric disease, diabetes, alcohol, and substance abuse, and forty-two patients with wrist or other fractures accompanying metacarpal fractures, were excluded. Following the file examination, patients with accompanying fractures, patients with open fractures, and patients who were operated on, were excluded from the study. The patients were classified using the 'propensity score matching' system. ‘Group 1’ who had a boxer’s fracture as a result of deliberately (as in a fight or anger causing punching a wall) punching; and ‘Group 2’ who had an accidental metacarpal fracture, and did not punch with aggression were selected as the control group (Figure 1).

**Figure 2:**
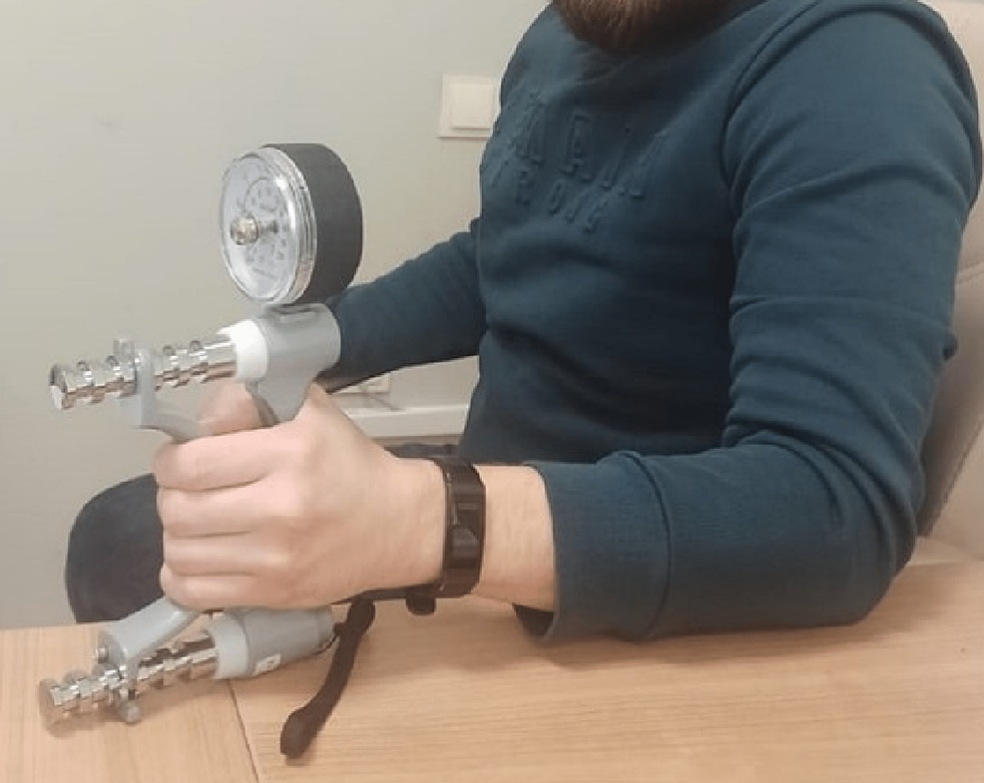
Grip force measurement with a Jamar dynamometer

All the patients were selected among those who had received conservative treatment. The Turkish validity of the Pain Catastrophizing Scale (PCS) questionnaire was utilized at least six weeks after fracture healing of the patients included in the study [13]. All patients were evaluated radiographically and clinically, which was performed after the sixth week. The quick ‘disabilities of the arm, shoulder, and hand’ (Q-DASH) score questionnaire was applied to all patients and the results were recorded (range: 0‑100, 0 as the best result) [14,15]. The patients’ Visual Analogue Scale (VAS) pain scores (range: 0‑10, 0 as the best result) were also evaluated and recorded. Grip strength was measured using a Jamar dynamometer (Asimow Engineering, Los Angeles, CA) with the elbow at 90 degrees of flexion and the forearm and wrist in neutral rotation (Figure 2). The data was recorded in kilograms.

The IBM SPSS version 26.0 software program (IBM Corp., Armonk, NY, USA) was used for statistical analysis. Median, interquartile range, and minimum and maximum values were used in the descriptive statistics of the data. The distribution of variables was measured with the Kolmogorov-Smirnov test. Since the quantitative data did not fit the normal distribution, we used the Mann-Whitney U test and presented the numerical data as the median (minimum-maximum). The Mann-Whitney U test was used in the analysis of quantitative independent data. The Chi-square test was used in the analysis of qualitative independent data, and the Fischer test was used when the chi-square test conditions were not met. The p-value of <0.05 was considered statistically significant.

## Results

Sixty-two individuals with regular follow-ups were selected for the study. A total of 62 patients injured between 2021 and 2022, comprised of 31 men with metacarpal fractures and 31 men with metacarpal fractures after an accident that occurred by throwing aggressive fists deliberately, were included in the study. Of the 62 fractures, 56 were 5th metacarpal and six were 4th metacarpal fractures.

The mean age of the patients was found to be 30.8 (18-50) in the 1st Group and 34.8 (18-64) in the 2nd Group, and no statistically significant difference was found (p=0.274). Since all patients were male, there was no significant difference in gender distribution between the groups. There was no statistically significant difference in fracture direction between the groups (p=0.291).

The main statistically significant outcome was in the PCS, VAS, and Q-DASH values; while the median PCS score was 10.5 (IQR: 12.3) for group 1, the median PCS score was 17.5 (IQR: 14.5) for group 2, and the PCS score was statistically significantly lower in group 1 (p=0.009). While the median VAS value was 0 (IQR: 0.3) for group 1, the median VAS value was 1 (IQR: 2.0) for group 2, and the VAS score was statistically significantly lower in group 1 (p<0.001). While the median Q-DASH value was 0 (IQR:2.3) for group 1, the median Q-DASH value was 3.4 (IQR:6.3) for group 2, and the Q-DASH score was statistically significantly lower in group 1 (p. =0.001).

The median value of grip strength measured by Jamar's hand dynamometer was 38.0 (IQR:5.3) on the boxer’s side and 39.0 (IQR:4.8) Kg on the side in group 2. No significant difference was observed between group 1 and group 2 in terms of grip strength values (p=0.815) (Table 1).

**Table 1:** Demographic and clinical information. All the variables do not fit the normal distribution and show the median values. The values in the cells represent the median, IQR, minimum and maximum values, respectively. Statistically significant findings are indicated in bold. PCS: Pain Catastrophic Scale, VAS: Visual Analogue Scale, DASH: Disabilities of The Arm, Shoulder, and Hand, Q-DASH: Quick Disabilities of The Arm, Shoulder, and Hand

	Boxer’s Fracture (Group 1) n=31	Other (Group 2) n=31	p-value
Age (Y)	25.5 (20.8) (18-50)	33.5 (19.5) (18-64)	0.274
Number of right metacarpal fracture	23	19	0.291
PCS	10.5 (12.3) (0-36)	17.5 (14.5) (7-34)	0.009
VAS	0 (0.3) (0-1)	1 (2) (0-2)	<0.001
DASH	11 (1) (11-13)	12.5 (3) (11-15)	0.001
Q-DASH	0 (2.3) (0-4.5)	3.4 (0) (0-9.1)	0.001
Grip Power	38 (5.3) (34-46)	39 (4.8) (30-46)	0.815

## Discussion

In this study, where we aimed to compare the Catastrophic Pain Scale and anxiety in boxer fractures and other hand fracture types, we found that PCS, VAS, and Q-DASH scores were lower in Boxer fractures.

Pain is a dominant determinant of post-treatment health status for upper extremity fractures [16]. In addition, the pain itself but also psychological factors may cause disabilities in chronic musculoskeletal patients [17]. For this reason, the way and the amount of pain perception vary from person to person and leads to clinical variations. This study demonstrated by the difference in PCS, VAS, and Q-DASH scores, that the psychological mechanisms associated with pain affect early functional recovery after conservative treatment of boxer fractures. In the literature, closed treatment of fifth metacarpal fractures with angulation less than 60-70 degrees has been shown to have high functional abilities with quick-DASH scores at four months [18]. Our results are similar to this study and accordingly, grip strength measurement did not show a significant difference.

Bot et al. have stated in their study that pain anxiety and catastrophic thoughts affect disability in patients recovering from injury [19], however, factors such as self-esteem and self-worth were not addressed, and more specifically, the higher the score on the pain disaster scale, the more dissatisfied the patient is found to be after recovery. Because of lower self-esteem and self-worth in individuals who injure themselves, such as in the case of a boxer’s fracture resulting from aggressive attitudes, the patient pretends to be satisfied regardless of the clinical functional outcome. Such patients should first communicate with the care providers and explain the significance of the situation, and the patient should be made to feel valued: psychological assistance should be sought if necessary.

In this regard, for example, cognitive behavioral therapy, exposure and acceptance therapy, and relaxation training are effective in improving coping strategies for chronic musculoskeletal conditions that result in a reduction in symptoms and disability [20]. We offer clinicians would receive help from the psychiatry department or psychological counseling when they encounter such patients would not only heal the fractures but would also help with earlier recovery, the fracture will heal at the same rate, and improve their quality of life.

Our study has several limitations. First of all, although all fractures in this study were treated conservatively, they were not homogeneous in terms of fracture classification, which may make a difference in clinical functional evaluation. However, our main aim was to show that some pain perception assessment systems can give different results in different patient groups, not on fracture healing. Our second limitation is the small number of patients: studies in larger series may provide a more comprehensive understanding.

## Conclusions

Boxer's fractures are the most common hand fractures, and most are treated conservatively. The functional outcome after fracture is satisfactory in most patients. However, only fracture follow-up may not be sufficient for every patient to meet the expectations of the patients and improve their clinical outcomes. Therefore, the etiology of patients presenting with a boxer’s fracture should be well understood, and if necessary, these patients should be treated multidisciplinary, with psychiatric help.

## References

[1] Gudmundsson P, Nakonezny PA, Lin J, Owhonda R, Richard H, Wells J (2021). Functional improvement in hip pathology is related to improvement in anxiety, depression, and pain catastrophizing: an intricate link between physical and mental well-being. BMC Musculoskelet Disord.

[2] Hove LM (1993). Fractures of the hand. Distribution and relative incidence. Scand J Plast Reconstr Surg Hand Surg.

[3] Hunter DJ, Buck R, Vignon E (2009). Relation of regional articular cartilage morphometry and meniscal position by MRI to joint space width in knee radiographs. Osteoarthritis Cartilage.

[4] van Onselen EB, Karim RB, Hage JJ, Ritt MJ (2003). Prevalence and distribution of hand fractures. J Hand Surg Br.

[5] Weum S, Millerjord S, de Weerd L (2016). The distribution of hand fractures at the university hospital of north Norway. J Plast Surg Hand Surg.

[6] Abdon P, Mühlow A, Stigsson L, Thorngren KG, Werner CO, Westman L (1984). Subcapital fractures of the fifth metacarpal bone. Arch Orthop Trauma Surg (1978).

[7] Ali A, Hamman J, Mass DP (1999). The biomechanical effects of angulated boxer's fractures. J Hand Surg Am.

[8] Birndorf MS, Daley R, Greenwald DP (1997). Metacarpal fracture angulation decreases flexor mechanical efficiency in human hands. Plast Reconstr Surg.

[9] Chung KC, Spilson SV (2001). The frequency and epidemiology of hand and forearm fractures in the United States. J Hand Surg Am.

[10] Osman A, Barrios FX, Gutierrez PM, Kopper BA, Merrifield T, Grittmann L (2000). The Pain Catastrophizing Scale: further psychometric evaluation with adult samples. J Behav Med.

[11] McCracken LM, Zayfert C, Gross RT (1992). The Pain Anxiety Symptoms Scale: development and validation of a scale to measure fear of pain. Pain.

[12] Rosenberger PH, Jokl P, Ickovics J (2006). Psychosocial factors and surgical outcomes: an evidence-based literature review. J Am Acad Orthop Surg.

[13] Süren M, Okan I, Gökbakan AM (2014). Factors associated with the pain catastrophizing scale and validation in a sample of the Turkish population. Turk J Med Sci.

[14] Gummesson C, Ward MM, Atroshi I (2006). The shortened disabilities of the arm, shoulder and hand questionnaire (QuickDASH): validity and reliability based on responses within the full-length DASH. BMC Musculoskelet Disord.

[15] Koldas Dogan S, Ay S, Evcik D, Baser O (2011). Adaptation of Turkish version of the questionnaire Quick Disability of the Arm, Shoulder, and Hand (Quick DASH) in patients with carpal tunnel syndrome. Clin Rheumatol.

[16] Souer JS, Lozano-Calderon SA, Ring D (2008). Predictors of wrist function and health status after operative treatment of fractures of the distal radius. J Hand Surg Am.

[17] Roh YH, Noh JH, Oh JH, Baek GH, Gong HS (2012). To what degree do shoulder outcome instruments reflect patients' psychologic distress?. Clin Orthop Relat Res.

[18] van Aaken J, Fusetti C, Luchina S (2016). Fifth metacarpal neck fractures treated with soft wrap/buddy taping compared to reduction and casting: results of a prospective, multicenter, randomized trial. Arch Orthop Trauma Surg.

[19] Bot AG, Mulders MA, Fostvedt S, Ring D (2012). Determinants of grip strength in healthy subjects compared to that in patients recovering from a distal radius fracture. J Hand Surg Am.

[20] Das De S, Vranceanu AM, Ring DC (2013). Contribution of kinesophobia and catastrophic thinking to upper-extremity-specific disability. J Bone Joint Surg Am.

